# Comparing the validity of the self reporting questionnaire and the Afghan symptom checklist: dysphoria, aggression, and gender in transcultural assessment of mental health

**DOI:** 10.1186/1471-244X-14-206

**Published:** 2014-07-18

**Authors:** Andrew Rasmussen, Peter Ventevogel, Amelia Sancilio, Mark Eggerman, Catherine Panter-Brick

**Affiliations:** 1 Fordham University, Dealy Hall 226, 441 East Fordham Rd, Bronx, NY 11215, USA; 2 UNHCR, Geneva, Switzerland; 3 Yale University, New Haven, CT, USA

**Keywords:** Validity, Assessment, Cultural concepts, Transcultural research, Idioms of distress, Aggression, Gender, Syndrome, Afghanistan

## Abstract

**Background:**

The relative performance of local and international assessment instruments is subject to ongoing discussion in transcultural research on mental health and psychosocial support. We examined the construct and external validity of two instruments, one developed for use in Afghanistan, the other developed by the World Health Organization for use in resource-poor settings.

**Methods:**

We used data collected on 1003 Afghan adults (500 men, 503 women) randomly sampled at three sites in Afghanistan. We compared the 22-item Afghan Symptom Checklist (ASCL), a culturally-grounded assessment of psychosocial wellbeing, with Pashto and Dari versions of the 20-item Self-Reporting Questionnaire (SRQ-20). We derived subscales using exploratory and confirmatory factor analyses (EFA and CFA) and tested total and subscale scores for external validity with respect to lifetime trauma and household wealth using block model regressions.

**Results:**

EFA suggested a three-factor structure for SRQ-20 - somatic complaints, negative affect, and emotional numbing - and a two-factor structure for ASCL - *jigar khun* (dysphoria) and aggression. Both factor models were supported by CFA in separate subsamples. Women had higher scores for each of the five subscales than men (p < 0.001), and larger bivariate associations with trauma (*r*s .24 to .29, and .10 to .19, women and men respectively) and household wealth (*r*s -.27 to -.39, and .05 to -.22, respectively). The three SRQ-20 subscales and the ASCL *jigar khun* subscale were equally associated with variance in trauma exposures. However, interactions between gender and *jigar khun* suggested that, relative to SRQ-20, the *jigar khun* subscale was more strongly associated with household wealth for women; similarly, gender interactions with aggression indicated that the aggression subscale was more strongly associated with trauma and wealth.

**Conclusions:**

Two central elements of Afghan conceptualizations of mental distress - aggression and the syndrome *jigar khun* – were captured by the ASCL and not by the SRQ-20. The appropriateness of the culturally-grounded instrument was more salient for women, indicating that the validity of instruments may be gender-differentiated. Transcultural validation processes for tools measuring mental distress need to explicitly take gender into account. Culturally relevant measures are worth developing for long-term psychosocial programming.

## Background

Expert guidelines advocate the inclusion of local perspectives of distress in the assessment of mental health in conflict-affected populations [1-3] to address issues of cultural relevance for psychosocial support programming [4-8]. Context-insensitive questionnaires may lead to inaccurate inferences concerning the needs of conflict-affected populations, whose members may or may not respond to mental health instruments in ways comparable to populations living in relatively secure communities [9]. Simply translating questionnaires into relevant languages is insufficient to address local conceptual issues [10-13]. Developing contextually valid emic (i.e., culturally-specific) instruments involves combining qualitative and quantitative approaches, marrying the ethnographic concern for local meanings with the epidemiological focus on replicable surveys [7,13,14]. In postconflict settings, mixed-methods techniques to develop and implement local measures have been applied to genocide-affected communities in Rwanda [15,16], refugees from Darfur in Chad [17], postpartum women in the Democratic Republic of Congo [18], violence-affected adults in Timor-Leste [19,20], internally displaced persons in Sri Lanka [21], and war-affected youth in several settings [22,23].

The Afghan Symptom Checklist (ASCL) was developed using ethnographic interviews and thematic coding of wellness and illness narratives to provide a culturally-grounded assessment of psychosocial wellbeing in Afghanistan [24]. In their study, Miller and colleagues described several Afghan idioms of distress: *asabi*, an anxiety-like state; *fishar*, or blood pressure abnormalities; and *jigar khun*, literally “liver blood,” an extreme and persistent dysphoria explained in humoral medicine as resulting from an overabundance of blood in the liver. The ASCL had good psychometric properties in a sample of 324 adults in Kabul.

While developing local measures like the ASCL has clear advantages in serving specific linguistic and cultural groups, it is not without costs. Considerable time and resources are needed to complete the essential steps of transcultural measure development: translate and back-translate content, evaluate content validity with international and local experts, and pilot instruments to evaluate psychometric properties [25]. Even when such steps are taken, there remain important questions concerning conceptual comparability. Results derived from measures specifically designed for one setting are often difficult to compare to results from others. In the face of such challenges, psychosocial programs often select screening instruments that have been previously implemented and proved reliable in other international settings. Such etic (i.e., culturally neutral) global mental health instruments are usually deployed to assess a range of symptoms indicative of common mental disorders, positing common factors in psychological distress. Typically these measures are used as screening instruments, and are not designed to be diagnostic of mental health disorders [26,27] or to provide within-population prevalence rates [9].

The Self-Reporting Questionnaire (SRQ-20) is one such instrument. The SRQ-20 was developed by the World Health Organization [3] to measure symptoms of common mental disorders across different cultures in low-income settings [28,29]. Designed for ease of administration, it consists of 20 items covering symptoms of anxiety and mood disorders with a binary (yes/no) response scale. The SRQ-20 has been assessed across a wide range of contexts, including conflict and refugee settings in Colombia [30], Guinea Bissau [31], Mozambique [32], Rwanda [33], Uganda [34], Iran [35], Pakistan [36], and Afghanistan [37,38]. In Afghanistan, the psychometric properties of a Pashto SRQ-20 were examined by Ventevogel and colleagues [39], who reported reasonable psychometric properties but large differences across gender. Some concern has been voiced that internationally-developed instruments such as the SRQ-20 may be insensitive to culturally-salient idioms of distress and thus may not differentiate between clinically-relevant and non-clinical distress [40].

We compared the ASCL and the SRQ-20 in a dataset from Afghanistan to examine the value of using a locally-developed emic measure versus using an internationally-developed etic measure. We approached this question methodologically in two stages: first we examined the construct validity of each measure by comparing items’ face validity and using factor analysis. Second, we compared the two measures’ external validity using regression models. In the latter we asked whether the measures were comparably associated with two third variables that have a well-known impact on mental health in low-income and conflict settings: exposure to traumatic events and socioeconomic status. Trauma and poverty are reliably associated with anxiety disorders, depression, and general emotional distress in conflict-affected adults [41-43]. Comparing how the ASCL and SRQ-20 were associated with these variables would provide an estimate of their external validity, a critical component of measurement validity [44]. A similar approach was used by Jayawickreme and colleagues to compare the PTSD Symptom Scale-Self Report [2] and the Beck Depression Inventory (BDI) to a locally-developed measure in a conflict affected sample in Sri Lanka [13]; the latter was found to explain additional variance in functional impairment.

In order to address the conceptual ambiguity inherent in using total scores calculated from multidimensional questionnaires, we combined construct and external validity goals to examine how empirically identified subscales on the ASCL and the SRQ-20 were differentially associated with trauma exposure and socioeconomic status. Our hypotheses were that certain subscales would draw on similar variance, but that the ASCL would include dimensions that the SRQ-20 did not, and these dimensions would account for more variance in external criteria. However, given that there has been little examination of the factor structure of either measure, we did not have specific hypotheses about the content of specific subscales or which subscale would be more associated with which criteria. Given the known gender segregation and disparities in mental health in Afghanistan [45-48], we tested hypotheses that these effects would be moderated by gender.

## Methods

### Sampling procedures

We drew our data from a gender-balanced community-based mental health survey undertaken in 2006 [37,38,49,50] in three research sites in central and northern Afghanistan (Kabul, Bamyan, and Mazar-e-Sharif). The study featured a school-based stratified random sample, beginning with a random sample of government schools with probability sampling proportional to size, and then a random sample of child-adult dyads (students and adult caregivers) targeting 320 dyads per site (on the basis of power calculations from pilot work). The research protocol was formally approved by the Ministry of Education in Afghanistan, the United Nations High Commissioner for Refugees in Peshawar, the ethics committees of the University of Peshawar and of Durham University, where the principal investigator received Welcome Trust funding for this research. In order to eliminate any risk that documented participation in research would result in danger to participants, informed consent was obtained verbally from all participants, and in writing only from school directors; only one potential participant refused to be interviewed.

### Interview and instrument preparation

We trained a small field team of male and female local researchers to interview participants in their preferred language, Dari or Pashto. The study featured two years of prior preparation in three sites (Wardak province, Kabul, and Peshawar) to achieve reliable translations, establish sampling and recruitment logistics, and conduct extensive pilots including test-retest reliability across sites. Translations from English to Dari and Pashto were undertaken by an Afghan clinical psychologist with professional experience in both the UK and Afghanistan, and independently checked by an Afghan psychologist at the University of Peshawar. We commissioned a professional translator and a linguist to undertake independent back-translations, which were then systematically reviewed for content validity by a panel comprised of fieldworkers and academics with expertise in anthropology, Middle Eastern studies, social work, child psychiatry, and cross-cultural psychology [37].

### Measures of trauma and socioeconomic status

Lifetime trauma exposures were measured using a checklist (20 yes/no items) adapted from the Harvard Trauma Questionnaire [35] previously implemented with adults in Afghanistan [37]. Our analyses focused on exposure severity (the total number of lifetime trauma reports) rather than specific types of traumatic experiences (detailed elsewhere [48]). To assess socioeconomic status, we asked participants to enumerate the total number of household goods from a specified list of 15 household items including cooking facilities, piped water, toilet, generator, sawdust-fuelled heater, mobile phone, radio, satellite dish, bicycle, and car. Such dimensional indices of household wealth are useful in contexts where self-reported income and expenditure data are likely unreliable.

### The self-reporting questionnaire

The SRQ-20 was developed as a mental health screening instrument for people presenting to general health services. Ventevogel and colleagues [39] reported that a Pashto version of SRQ-20, implemented in the eastern Afghan province of Nangarhar, revealed a two-factor structure consisting of “common disorders” and “social disability.” Compared with a structured psychiatric interview, the SRQ-20 had moderate sensitivity and specificity for predicting the presence of common mental health disorders.

### The Afghan symptom checklist

The ASCL consists of 22 items drawn from thematic coding of transcripts of ethnographic interviews undertaken with adult men and women in Kabul [24]. Among items are three Dari terms representing Afghan idioms of distress: *jigar khun*, a term describing “a form of sadness that includes grief following interpersonal loss but that may also be a reaction to any deeply disappointing or painful experience” (p. 425); *asabi*, a term for “feeling nervous or highly stressed” (p. 425), used to describe a state of nervous agitation when overwhelmed by stress; and *fishar*, or “pressure” locally thought to map onto blood pressure variation. While developing the ASCL, Miller and colleagues combined the idioms *fishar-e-bala* (high blood pressure) and *fishar-e-payin* (low blood pressure) into one item, but after factor analysis recommended that they be separated (footnote 3, p.426). The ASCL had adequate reliability and a latent variable structure with three interpretable factors: “sadness with social withdrawal and somatic distress” (p. 425); “ruminative sadness without social isolation and somatic distress” (p. 425); and stress-induced reactivity, consisting of quarreling, beating one’s children, and *asabi*.

### Analytic strategy

To examine face validity, we compared SRQ-20 items with ASCL items qualitatively, identifying parallel content and item differences. To examine construct validity, we followed a sequence of fitting exploratory and confirmatory factor models. We split the full sample into three subsamples, using simple random selection to select a random third of cases for an exploratory subsample followed by a random half of the unselected subsample to select two confirmatory subsamples. All selections were made using the “select cases” option in the Data menu of SPSS. We then used EFA with varimax rotation to suggest confirmatory models, and confirmatory factor analysis (CFA) to test these models in the remaining two subsamples. Because of the arbitrariness of using eigenvalues greater than 1.0 to suggest the number of latent factors, we examined scree plots and rotated factor plots in EFA to build interpretable confirmatory models. We operationalized meaningful factor loadings as > 0.400. We ran the EFA using both SPSS and Mplus, using the former for continuous responses on the ASCL and the latter for binary responses on the SRQ-20 [36]; we estimated CFA models using MPlus only. We qualitatively compared the content of factors across measures, with interpretation aided by reference to extant global mental health literature, including previous work in Afghanistan [24,51].

We tested external validity by examining the value of adding ASCL scores following SRQ-20 scores to regression block models predicting trauma and wealth indices. We conducted these analyses for (1) total scores as a dimensional measure of mental health problems, and (2) subscale scores for each ASCL subscale following each SRQ-20 subscale. Relative advantages in external validity of the ASCL over the SRQ-20 were operationalized as statistically significant beta coefficients of ASCL scores in regression models that included SRQ-20 scores in previous blocks. We also examined how these associations might be moderated by gender by including interactions between gender and ASCL subscales. We entered gender in the first block, SRQ-20 scores in the second block, ASCL scores in the third, and interactions between gender and ASCL scores in the fourth.

## Results

### Sample demographics

Our sample included 1003 adults (500 men, 503 women), averaging 36.44 years (SD 12.37) of age. A third were from the Kabul area (n = 364, 36.3%), a third from Bamyan (n = 327, 32.6%), and a third from Mazar-e-Sharif (n = 311, 31.0%). The sample was largely Tajik (n = 429, 42.8%) and Hazara (n = 424, 42.3%), with small minorities of Pashtun (n = 86, 8.6%), Uzbek (n = 39, 3.9%) and other ethnicities (n = 24, 2.4%). Half (n = 501, 50.0%) were uneducated, just over a third (n = 352, 35.1%) had some primary or secondary education, and ten percent (n = 101, 10.1%) had some post-secondary education (n = 48, 4.8% were missing education data). Just under half (n = 495, 49.4%) were employed (49, 49% were missing employment data). The three subsamples used for exploratory and confirmatory factor analyses did not differ by gender, age, ethnicity, region, education or employment. The mean score for the SRQ-20 was 13.03 (SD 2.52); there were large differences between women (9.45, SD 4.51) and men (5.51, SD 3.75; p < 0.0001; d 0.95). The mean score for the ASCL was 35.21 (SD 5.72), with higher scores for women (52.44, SD 15.73) than men (38.04, SD 10.99; p < 0.0001; d 1.06).

### Review of SRQ-20 and ASCL

We identified five comparable items on the SRQ-20 and the ASCL. These included SRQ1, “headaches” and ASCL11, “had a headache”; SRQ2, “poor appetite” and ASCL2 “lack of appetite”; SRQ3, “bad sleep” and ASCL3, “difficulty falling asleep”; SRQ9, “unhappy” and ASCL9, “felt sad”; and SRQ10, “cry more than usual” and ASCL1, “cried”. Three other SRQ items were broadly comparable to ASCL items: SRQ4, “frightened easily” and ASCL14, “felt startled”; SRQ6, “nervous, tense, worried” and ASCL17, “experienced *asabi*”; and SRQ8, “trouble thinking clearly” and both ASCL23, “trouble concentrating” and ASCL16, “thinking too much”. The comparison of other items showed that SRQ-20 items included more somatic complaints, while the ASCL included items related to aggression. Following comparison of item content, we removed the ASCL item “difficulty meeting responsibilities because of *jigar khun*” from subsequent analyses in order to avoid conflating emotional distress with functional impairment.

### SRQ-20 factor analyses

Table 1 presents the 3-factor EFA for the SRQ-20 items from the exploratory sample. The first factor consisted primarily of somatic complaints, and was dominated (loading > .800) by the item “poor appetite”. The second factor was best characterized as general negative affect; it was dominated by “cry more than usual” and notably included “thoughts of ending your life” and four items that also loaded on the first factor. The third factor consisted of difficulty making decisions and loss of pleasure, suggesting emotional numbing. The 3-factor model somatic complaints, negative affect, and emotional numbing fit the two confirmatory samples reasonably well (CFI = .918, TLI = .946, RMSEA = .061 in confirmatory sample 1; CFI = .929, TLI = .947, RMSEA = .056 in confirmatory sample 2).

**Table 1:** Exploratory factor analysis (varimax rotation) of the self-reported questionnaire (SRQ-20) items

	**Somatic complaints**	**Negative affect**	**Emotional numbing**
Poor appetite (SRQ2)	0.833	-	-
Poor digestion (SRQ7)	0.735	-	-
Headaches (SRQ1)	0.617	-	-
Uncomfortable feelings in your stomach (SRQ19)	0.606	-	-
Feel tried all the time (SRQ18)	0.537	0.511	-
Bad sleep (SRQ3)	0.501	0.431	-
Easily tired (SRQ20)	0.449	0.631	
Hands shake (SRQ5)	0.404	0.598	-
Cry more than usual (SRQ10)	-	0.802	-
Thoughts of ending your life (SRQ17)	-	0.633	-
Feel worthlessness (SRQ16)	-	0.604	0.480
Nervous, tense, worried (SRQ6)	-	0.586	-
Frightened easily (SRQ4)	-	0.552	-
Unhappy (SRQ9)	-	0.473	0.612
Trouble thinking clearly (SRQ8)	-	0.319	-
Difficulty making decisions (SRQ12)	-	-	0.825
Difficulty enjoying activities (SRQ11)	-	-	0.774
Unable to play a useful part in life (SRQ14)	-	-	0.665
Lost interest in things (SRQ15)	-	-	0.572
Daily work suffering (SRQ13)	-	-	0.561

### ASCL factor analyses

During EFA we found that the ASCL item “quarreling with a neighbor or friend” was weakly associated with all other items, constituted its own factor in initial EFA solutions, and was a clear outlier in rotated factor plots. We dropped this item from subsequent analyses. Table 2 presents the 3-factor EFA model for ASCL items. The first factor (16 items) consisted of symptoms of negative affect (e.g., “felt sad”), depressed mood (e.g., “felt hopeless”) and nervous agitation (“*asabi”*). Because of the dominance of “became *jigar khun”* (loading > .800) and item concordance with Miller and colleagues’ description of the syndrome *jigar khun*, we labeled the first factor *jigar khun*. The second factor (four items) was comprised of aggressive behavior toward family members and self; it also included “cried,” which also loaded with the first factor. The third factor consisted of just two inversely-related items: “*fishar-e-bala”* (loading positively) and “*fishar-e-payin”* (loading negatively). We named this factor *fishar*. The 3-factor model suggested by the EFA fit the two confirmatory samples adequately (CFI = .943, TLI = .974, RMSEA = .087 in confirmatory sample 1; CFI = .920, TLI = .959, RMSEA = .099 in confirmatory sample 2). Although RMSEA statistics were higher than the recommended .06, both CFI and TLI suggested good fit [52]. Varying the model slightly (e.g., not allowing for double-loading items) did not result in improved fit.

**Table 2:** Exploratory factor analysis (varimax rotation) of the Afghan symptom checklist (ASCL) items

	***Jigar Khun***	**Aggression**	***Fishar***
Felt sad (ASCL9)	0.823	-	-
Become *jigar khun* (ASCL10)	0.815	-	-
Thinking too much (ASCL16)	0.778	-	-
Trouble concentrating (ASCL23)	0.774	-	-
Experienced *asabi* (ASCL17)	0.710	-	-
Had bad memories (ASCL15)	0.641	-	-
Felt hopeless (ASCL6)	0.608	-	-
Felt irritable (ASCL13)	0.593	-	-
Had a headache (ASCL11)	0.588	-	-
Felt startled (ASCL14)	0.588	-	-
Trouble remembering things (ASCL18)	0.572	-	-
Cried (ASCL1)	0.522	0.466	-
Isolated oneself socially (ASCL8)	0.501	-	-
Had a nightmare (ASCL12)	0.483	-	-
Lack of appetite (ASCL2)	0.477	-	-
Difficulty falling asleep (ASCL3)	0.406	-	-
Beaten or hurt yourself (ASCL19)	-	0.733	-
Quarrel with family member (ASCL4)	-	0.726	-
Beat someone in family (ASCL7)	-	0.661	-
Felt *fishar-e- bala* (ASCL20)	-	-	0.628
Felt *fishar-e- payin* (ASCL21)	-	-	−0.582

Two items were removed from consideration: “quarreling with a neighbor or friend” (ASCL5) and “difficulties meeting responsibilities because of *jigar khun*” (ASCL22; see text).

### Comparing SRQ-20 and ASCL subscales in the full sample

Because of comparable fit across subsamples we used the full sample for subsequent analyses. The SRQ-20 subscales were constructed based on the 3-factor solution. They attained good Cronbach’s α reliability (mathematically identical to the Kruder-Richardson’s reliability coefficient for binary responses [53]), as follows: somatic complaints (8 items) α = .78, negative affect (11 items) α = .77, and emotional numbing (7 items) α = .74. For ASCL, we retained two subscales, *jigar khun* and aggression, and eliminated the third, *fishar*. As a subscale, the two-item *fishar* would have been unstable [54], as shown by the sum of the item variances being greater than the scale variance, resulting in a negative Cronbach’s α of -.23. The *jigar khun* and aggression subscales attained satisfactory Cronbach’s α reliability coefficients: *jigar khun* (16 items) α = .91, and aggression (4 items) α = .66.

Mean scores for SRQ-20 subscales were: somatic complaints 3.93 (SD 2.46), negative affect 4.38 (SD 2.76), and emotional numbing 1.68 (SD 1.83). On all SRQ-20 subscales, women scored higher than men (p < 0.001) and two of the three differences were large: women’s somatic complaints score averaged 4.90 (SD 2.21) and men’s 2.95 (SD 2.30), d 0.86; their negative affect score averaged 5.71 (SD 2.62) and men’s 3.04 (SD 2.18), d 1.11; their emotional numbing score averaged 1.96 (SD 2.09) and men’s 1.40 (SD 1.47), d .31. Mean scores for ASCL subscales were as follows: *jigar khun*, 32.49 (SD 12.12), and aggression, 5.72 (SD 2.52). On both subscales, women showed higher symptom scores (p < 0.001) and effect size differences were large (d > .7): women’s *jigar khun* score averaged 38.17 (SD 12.27) and men’s 26.77 (SD 8.83), d 1.07; their aggression score averaged 7.03 (SD 2.86) and men’s 4.41 (SD 1.03), d 1.22.

Table 3 shows the associations between SRQ-20 and ASCL subscales and variables of interest. Correlations between subscales in the full sample were large (range .39 to .80); removing double-loading items from subscales did not substantially alter correlations (range .38 to .72). We report full subscales (i.e., with double-loading items allowed) here. Both trauma exposures and wealth were significantly correlated across the ASCL and SRQ-20 subscales for women, but inconsistently so for men.

**Table 3:** Correlation between Afghan symptom checklist (ASCL) and self-reporting questionnaire (SRQ-20) subscales and lifetime trauma exposure and household wealth (n = 1003)

		**Afghan symptom checklist**		**Self-reporting questionnaire**		
		***Jigar Khun***	**Aggression**	**Somatic complaints**	**Negative affect**	**Emotional numbing**
Lifetime trauma exposure	Total	0.17*	0.10*	0.13*	0.17*	0.17*
	Men	0.18*	0.01	0.10	0.19*	0.10
	Women	0.28*	0.25*	0.26*	0.29*	0.24*
Wealth index	Total	- 0.11*	- 0.06	- 0.22*	- 0.14*	- 0.18*
	Men	- 0.11*	0.06	- 0.22*	- 0.18*	- 0.12*
	Women	- 0.28*	- 0.27*	- 0.39*	- 0.31*	- 0.27*

* = p < 0.01.

### Comparing external validity of ASCL and SRQ-20 scores

We found strong and consistent associations with gender and SRQ-20 scores (Total scores, Table 4). For trauma, we found no independent associations with ASCL scores or with the gender-by-ASCL interaction (total R^2^ = 0.077). For household wealth, associations with ASCL scores and gender-by-ASCL were significant, over and above associations with SRQ-20 (total R^2^ = 0.117).

**Table 4:** External validity of Afghan symptom checklist (ASCL) given the self-reported questionnaire (SRQ-20) subscales, using the total items in each scale (n = 1003)

**Dependent**	**Model**	**B coefficient**	**SE**	**Beta**	**T**	***p value***	**Δ R**^**2**^
Trauma events	Constant	5.371	0.942		5.702	0.000	
	Gender	−1.903	0.583	−0.356	−3.262	0.001	0.008
	SRQ-20 Total scores	0.116	0.027	0.199	4.271	0.000	0.062
	ASCL Total scores	−0.005	0.024	−0.030	−0.217	0.828	0.006
	Gender × ASCL	0.016	0.013	0.239	1.215	0.225	0.001
Wealth index	Constant	1.730	1.097		1.577	0.115	
	Gender	3.643	0.679	0.572	5.364	0.000	0.025
	SRQ-20 Total scores	−0.221	0.032	−0.319	−7.012	0.000	0.086
	ASCL Total scores	0.063	0.028	0.305	2.296	0.022	0.000
	Gender × ASCL	−0.040	0.015	−0.515	−2.677	0.008	0.006

Δ R^2^ represents change in R^2^ value.

**Figure 1:**
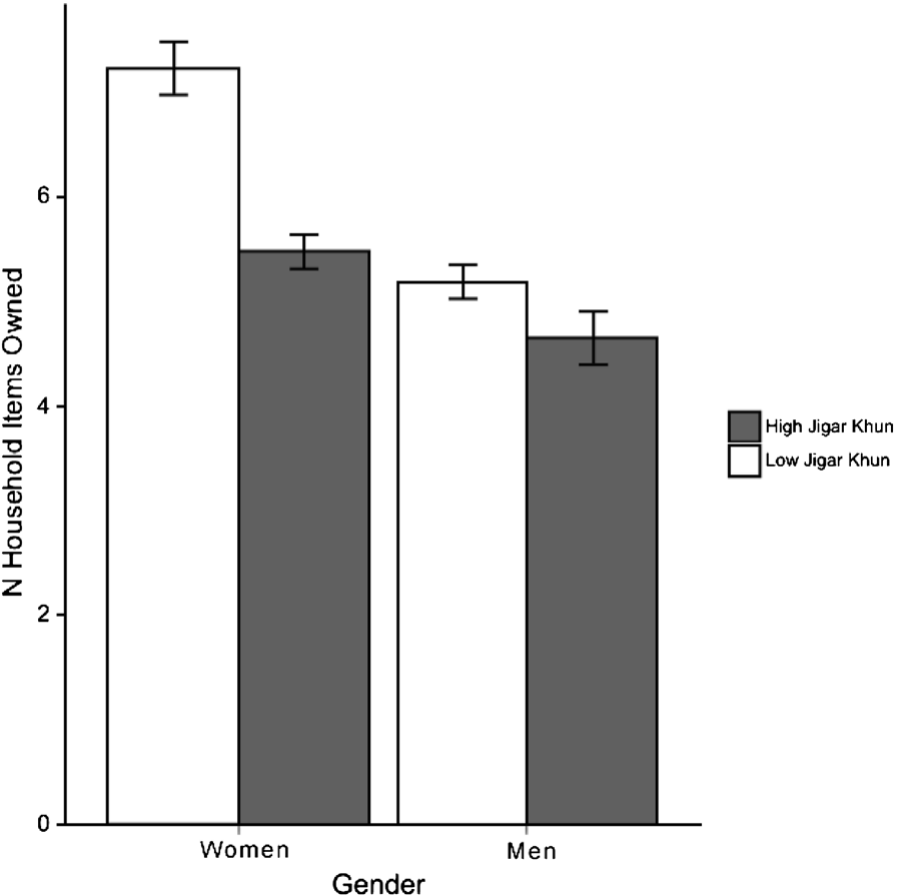
**Afghan symptom checklist (ASCL) *jigar khun *** **subscale was associated with wealth indices for women only.**

Results for the *jigar khun* ASCL subscale are shown in Table 5. For trauma, neither *jigar khun* nor the gender-by-*jigar khun* interactions were associated with variance beyond the SRQ-20 subscales of somatic complaints, negative affect, or emotional numbing. For wealth, both *jigar khun* and gender-by-*jigar khun* were associated with variance with respect to the somatic complaints SRQ-20 subscale, but not with the negative affect or emotional numbing SRQ-20 subscales. The interaction between gender and *jigar khun* scores is presented graphically in Figure 1. *Jigar khun* was negatively associated with wealth only for women, with women above median *jigar khun* scores reporting fewer household items.

**Table 5:** **External validity of the *Jigar Khun *** **subscale of the Afghan symptom checklist (ASCL) given the self-reported questionnaire (SRQ-20) subscales (n = 1003)**

**Dependent**	**Model**	**B**	**SE**	**Beta**	**t**	***p*** **value**	**Δ R**^**2**^
Trauma events	(Constant)	4.772	0.832		5.738	0.000	
	Gender	−1.561	0.530	−0.292	−2.946	0.003	0.008
	SRQ-20 Somatic complaints	0.069	0.047	0.064	1.464	0.144	0.031
	*ASCL Jigar Khun*	0.027	0.030	0.125	0.928	0.354	0.024
	Gender × *Jigar Khun*	0.012	0.015	0.150	0.805	0.421	0.001
Trauma events	(Constant)	5.048	0.818		6.171	0.000	
	Gender	−1.686	0.521	−0.315	−3.235	0.001	0.008
	SRQ-20 Negative Affect	0.186	0.046	0.191	4.032	0.000	0.061
	*ASCL Jigar Khun*	0.009	0.029	0.040	0.310	0.757	0.008
	Gender × *Jigar Khun*	0.012	0.015	0.145	0.793	0.428	0.001
Trauma events	(Constant)	4.682	0.809		5.785	0.000	
	Gender	−1.468	0.516	−0.274	−2.843	0.005	0.008
	SRQ-20 Emotional Numbing	0.149	0.050	0.101	2.965	0.003	0.033
	*ASCL Jigar Khun*	0.029	0.028	0.129	1.027	0.305	0.030
	Gender × *Jigar Khun*	0.011	0.015	0.135	0.737	0.461	0.001
Wealth index	(Constant)	1.696	0.957		1.773	0.077	
	Gender	3.696	0.610	0.580	6.063	0.000	0.025
	SRQ-20 Somatic Complaints	−0.441	0.054	−0.340	−8.100	0.000	0.093
	*ASCL Jigar Khun*	0.095	0.034	0.363	2.801	0.005	0.000
	Gender × *Jigar Khun*	-.055	0.017	−0.574	−3.190	0.001	0.009
Wealth index	(Constant)	2.662	0.965		2.758	0.006	
	Gender	3.071	0.615	0.483	4.994	0.000	0.024
	SRQ-20 Negative Affect	-.272	0.054	−0.236	−5.003	0.000	0.061
	*ASCL Jigar Khun*	.043	0.034	0.162	1.257	0.209	0.003
	Gender × *Jigar Khun*	-.035	0.017	−0.361	−1.992	0.047	0.004
Wealth index	(Constant)	3.163	0.955		3.312	0.001	
	Gender	2.747	0.609	0.431	4.509	0.000	0.025
	SRQ-20 Emotional Numbing	−0.256	0.059	−0.147	−4.326	0.000	0.042
	*ASCL Jigar Khun*	0.018	0.033	0.068	0.540	0.589	0.017
	Gender × *Jigar Khun*	−0.034	0.017	−0.354	−1.955	0.051	0.004

Δ R^2^ represents change in R^2^ value.

**Figure 2:**
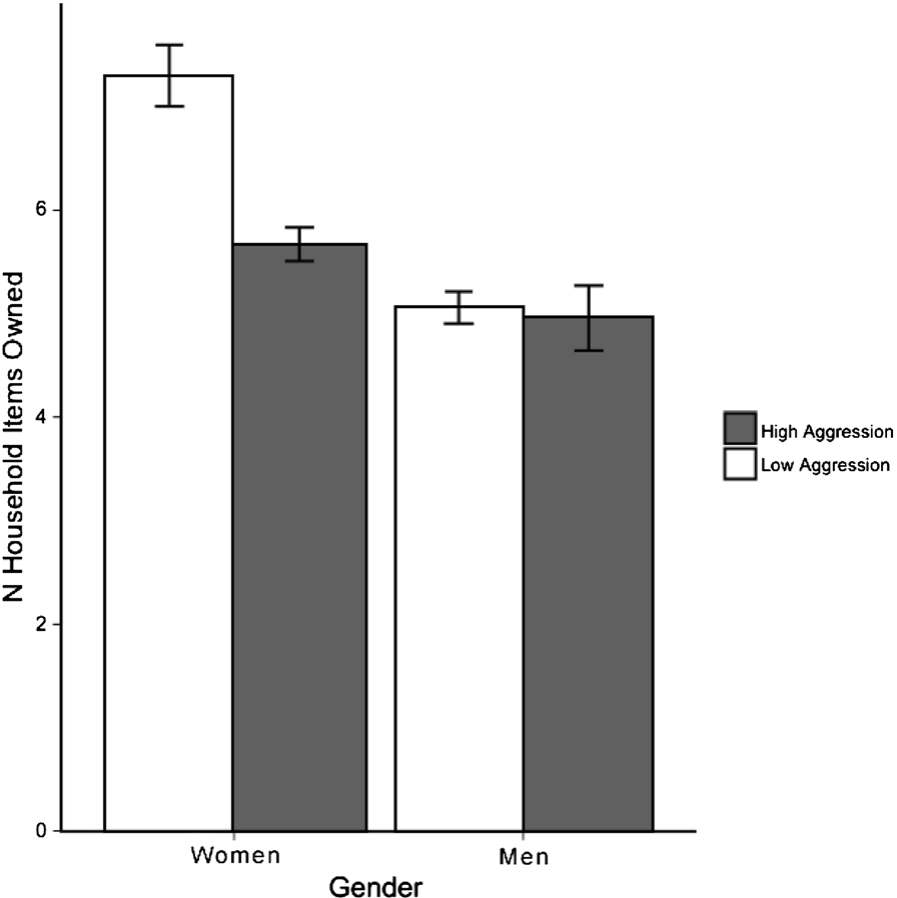
Afghan Symptom Checklist (ASCL) aggression subscale was associated with wealth indices for women only.

We repeated this analysis for the aggression ASCL subscale (Table 6). Gender and SRQ-20 subscales showed strong and consistent associations with trauma and wealth. ASCL aggression and the gender-by-aggression interaction were weakly significant (.05 > *p* > .01) in these regression models. In contrast, they were strongly and consistently significant for wealth. ASCL aggression and gender-by-aggression were associated with variance in wealth above and beyond the SRQ-20 subscales somatic complaints, negative affect, and emotional numbing. The interaction between gender and ASCL aggression scores is presented graphically in Figure 2. Similar to results for *jigar khun*, women in poorer households reported more aggressive behaviors.

**Table 6:** External validity of the Aggression subscale of the Afghan symptom checklist (ASCL) given the self-reported questionnaire (SRQ-20) subscales (n = 1003)

**Dependent**	**Model**	**B**	**SE**	**Beta**	**t**	***p*** **value**	**Δ R**^**2**^
Trauma events	(Constant)	6.730	1.069		6.293	0.000	
	Gender	−2.392	0.603	−0.447	−3.969	0.000	0.008
	SRQ-20 Somatic Complaints	0.166	0.039	0.152	4.250	0.000	0.031
	ASCL Aggression	−0.328	0.231	−0.309	−1.419	0.156	0.014
	Gender × Aggression	0.255	0.120	0.605	2.122	0.034	0.004
Trauma events	(Constant)	6.777	1.058		6.403	0.000	
	Gender	−2.474	0.595	−0.463	−4.155	0.000	0.008
	SRQ-20 Negative Affect	0.239	0.038	0.246	6.323	0.000	0.061
	ASCL Aggression	−0.358	0.228	−0.337	−1.567	0.117	0.005
	Gender x Aggression	0.239	0.119	0.566	2.012	0.044	0.004
Trauma events	(Constant)	6.341	1.070		5.928	0.000	
	Gender	−1.980	0.598	−0.370	−3.310	0.001	0.008
	SRQ-20 Emotional Numbing	0.199	0.049	0.136	4.045	0.000	0.033
	ASCL Aggression	−0.239	0.230	−0.225	−1.041	0.298	0.013
	Gender x Aggression	0.209	0.120	0.494	1.738	0.082	0.003
Wealth index	(Constant)	−0.250	1.219		−0.205	0.837	
	Gender	4.714	0.687	0.740	6.863	0.000	0.025
	SRQ Somatic Complaints	−0.412	0.044	−0.318	−9.276	0.000	0.093
	ASCL Aggression	0.971	0.264	0.768	3.682	0.000	0.004
	Gender x Aggression	−0.565	0.137	−1.126	−4.127	0.000	0.015
Wealth index	(Constant)	0.090	1.243		0.073	0.942	
	Gender	4.336	0.699	0.682	6.202	0.000	0.024
	SRQ-20 Negative Affect	−0.292	0.044	−0.253	−6.583	0.000	0.061
	ASCL Aggression	0.830	0.268	0.657	3.095	0.002	0.004
	Gender × Aggression	−0.491	0.139	−0.979	−3.522	0.000	0.011
Wealth index	(Constant)	0.677	1.254		0.540	0.589	
	Gender	3.689	0.701	0.579	5.260	0.000	0.025
	SRQ-20 Emotional Numbing	−0.288	0.058	−0.165	−5.004	0.000	0.042
	ASCL Aggression	0.693	0.269	0.548	2.573	0.010	0.009
	Gender × Aggression	−0.450	0.141	−0.895	−3.197	0.001	0.009

Δ R^2^ represents change in R^2^ value.

## Discussion

Strengths of the current study include an explicit focus on the cultural relevance of instruments, two years of preparation and pilot surveys, recruitment across multiple sites, random sampling, and gender balance. We feel that because of these strengths this study avoids many of the limitations common in the field. However, like all research projects, this study is not without its limitations. These include not measuring functional impairment, relatively low levels of associations between distress scores and external criterion variables, double-loading items in EFA models, and suboptimal RMSEA statistics in CFA models for the ASCL. Nevertheless, we believe our analyses comparing the external validity of the SRQ-20 and the ASCL are clinically meaningful, and we encourage researchers to build upon these findings to improve mental health assessment in Afghanistan and other conflict-affected settings.

Our findings suggest that the ASCL was a better measure of distress than the SRQ-20 for women, while the two measures were similar for men. Given the strict gender demarcation of Afghan society [24,48,51,55,56], we expected to find striking gender differences. Men and women inhabit different social and emotional spaces in Afghanistan, with downstream implications for psychosocial [37,46-48] and physiological [50] wellbeing. The large mean differences across ASCL and SRQ-20 subscale scores are thus consistent with the literature. Why the cultural appropriateness of the ASCL seemed more salient for women is less clear. Being involved in the public sphere, men may be more likely to recognize the more generalized modes of expression codified in the SRQ-20, while women, who have limited social interactions outside the home, may respond to more culturally-grounded expressions of distress. It may also be that responses follow the script of gender-differentiated display rules (i.e., culture of emotions). Permissible ways to express distress are gendered, such that Afghan men may publicly express anger, jealousy, and hate, but not fear, grief, or doubt – emotions that might bring shame to families’ honor [57]. Thus in our data, men’s scores may have been constrained by a ceiling effect on particular item responses.

The hypothesis that using total scores would mask important variability with implications for external validity was supported by our findings. The internal structures we found for the SRQ-20 and the ASCL were somewhat distinct from previous descriptions. The latent structure of the SRQ-20 included three factors: somatic complaints, negative affect, and emotional numbing. Although different from the two-factor solution identified by Ventevogel and colleagues [39] using a different Afghan dataset, this structure still largely mirrors symptom clusters of common mental disorders – i.e., anxiety and depression. Unlike the factor structure reported by Miller and colleagues [24], the ASCL in our data was comprised of a latent variable structure consisting of *jigar khun*, aggression, and *fishar. Jigar khun* was by far the dominant latent variable in exploratory and confirmatory models of ASCL scores, suggesting that it represents a specific cultural syndrome rather than simply an idiom of distress. Idioms of distress are more general ways of experiencing and expressing distress within specific local contexts [58-60], whereas cultural syndromes (as described by Nichter) are “widely recognized prototypical cultural ailment[s] that encompasses a fuzzy set of associations coalescing around one or more core cultural symbols” (p.407) [58]. In prior development of the ASCL [24], the item *jigar khun* consisted primarily as grief. In our study, the inclusion of *asabi* and other items representing states of arousal within the *jigar khun* subscale seems to suggest that *jigar khun* is a multidimensional construct, combining several symptoms of common mental disorders. In other words *jigar khun* is more than a general expression of distress, and we feel that it meets criteria as an Afghan cultural syndrome.

*Jigar khun* was not associated with variance in trauma events beyond negative affect and emotional numbing as measured by the SRQ-20. This suggests that *jigar khun* pulls on similar sources of variance as somatic complaints, negative affect, and emotional numbing for Afghans with respect to trauma. With respect to socioeconomic status, however, the models for *jigar khun* and SRQ-20 somatic subscale suggest a different conclusion – namely, that *jigar khun* is associated with household wealth above and beyond somatic complaints for women. That dysphoria is associated with poverty in populations affected by armed conflict is well-known [41,42]. What our findings seem to suggest is that this association may be gender-dependent. Further research should examine the gendered nature of features of distress, especially in regions with strict gender segregation such as Afghanistan.

In contrast to *jigar khun*, we found that aggression was associated with both trauma and socioeconomic status above and beyond the SRQ-20 subscales, especially for women. This suggests that the ASCL is a better measure of Afghan distress in large part because it includes aggression items. Aggression has been for the most part ignored in standard North American and European psychiatric concepts of common mental health disorders. Its prominence here is consistent with reports that behavioral disturbances are common in other taxonomies of mental distress. Explosive anger is a critical element of psychosocial distress among West Papuan refugees in Australia [61]. In Timor-Leste, explosive anger is associated with ongoing socio-economic disadvantage [43] and past trauma exposure [20]. Rees and colleagues [62] have emphasized the risk of explosive anger for women in particular, who experience the disproportionate impact of poverty hardships and human rights abuses. In Afghanistan, Catani, Schauer and Neuner [63] have linked war exposure for adults and increased levels of abusive behavior toward their children. Our findings show that aggression is an important dimension of Afghan expressions of distress, and that it can take the form of self-directed as well as interpersonal violence. In content analyses of qualitative interview data collected concurrently with the present study [48] and in other work conducted in Afghanistan [45], women with intense emotional distress reported hitting themselves, beating their children, and causing injury to others. Indeed, the connection between aggressive behavior and psychological distress in Afghanistan is reflected in language used to describe distress. In Pashto and Dari, the word *khapa* means angry and annoyed as well as sad, anxious, and worried. The word *khapgan* (lit: hardship) refers to either sadness or anger, depending on social context [64].

Our findings are specific to comparing measurement across constructs, and do not imply equivalence of meaning. In other words, that the subscales of the SRQ-20 and the *jigar khun* subscale of the ASCL drew upon a similar pool of variance *psychometrically* does not necessarily imply that the SRQ-20 subscales represent culturally meaningful concepts. Being associated with similar variance in external criteria may imply comparable content and external validity, but does not imply similar construct validity. This is not to undermine those who would argue that the SRQ-20 is useful for screening in humanitarian response efforts; indeed, our findings suggest that it is, insofar as it is associated with important variance in Afghan emotional distress. However, presenting evidence of psychometric association is not the same as presenting evidence of culturally-grounded parallel meanings. Symptom checklists and screening inventories are situated within the cultures of their development; useful in comparative monitoring and evaluation, they are rarely designed to capture the subtleties of local explanatory models.

Studies that have compared locally-developed, emic mental health measures to globally-deployed, etic measures suggest considerable psychometric overlap between instruments, but highlight that local development does provide some additional value in terms of both content and external validity [12,21]. Findings from the current study echo this conclusion. Although the body of literature is still relatively small, the consistent advantage of locally-developed measures suggests that efforts to develop them are indeed worthwhile. However, the equally consistent finding regarding the large overlap in content and external validity suggests that decisions not to develop culturally-grounded instruments may be justified, depending on project scope and resources. We are reminded of the emphasis placed by Kohrt and colleagues’ [8] on the consideration of research purpose as key to tool selection. Where the goals of psychosocial programming in emergency contexts are short-term and relatively limited in scope, resources dedicated to developing local measures to screen for mental health are perhaps better spent elsewhere. Over the long term, however, developing culturally-grounded and locally validated emic measures will significantly improve the quality of psychosocial programming, as the small but consistent advantages of emic measures observed in our findings and others’ are likely magnified over time. For sustainable programs it is particularly important that case-identification and monitoring and evaluation tools capture salient expressions of distress [65]. In terms of research, the development of emic instruments is critical because the goals of methodological excellence and cultural specificity overshadow many other possible concerns. Using such scales alongside etic scales may open up a range of strategies to examine multiple health outcomes with multiple, complementary tools. Psychosocial programs deciding whether or not to devote the resources necessary to develop local measures of distress where there are none should weigh the purposes of the tool and the potential for long-term engagement.

## Conclusions

Comparing the ASCL and SRQ-20 revealed substantial overlap in construct and external validity, but the ASCL captured more variance associated with trauma and poverty for women. These findings highlight the advantages of locally-developed measures of mental health and the importance of considering gender in transcultural validation. In addition, practitioners should not ignore the role of aggression in emotional distress in cross-cultural setting. Culturally relevant measures are worth developing for long-term psychosocial programming.

## Competing interests

The authors declare that they have no competing interests.

## Authors’ contributions

AR was responsible for the analytic plan, with help from PV and AS. CPB was the primary investigator on the original survey, undertaken with ME and local collaborators (no other authors were involved in data collection). All authors contributed to authorship of this paper.

## Pre-publication history

The pre-publication history for this paper can be accessed here:

http://www.biomedcentral.com/1471-244X/14/206/prepub
